# Reliability and generalization of gait biometrics using 3D inertial sensor data and 3D optical system trajectories

**DOI:** 10.1038/s41598-022-12452-6

**Published:** 2022-05-19

**Authors:** Geise Santos, Tiago Tavares, Anderson Rocha

**Affiliations:** 1 Artificial Intelligence Lab. (Recod.ai), Institute of Computing, University of Campinas, Campinas, Brazil; 2 School of Electrical and Computer Engineering, University of Campinas, Campinas, Brazil

**Keywords:** Computational science, Computer science

## Abstract

Particularities in the individuals’ style of walking have been explored for at least three decades as a biometric trait, empowering the automatic gait recognition field. Whereas gait recognition works usually focus on improving end-to-end performance measures, this work aims at understanding which individuals’ traces are more relevant to improve subjects’ separability. For such, a manifold projection technique and a multi-sensor gait dataset were adopted to investigate the impact of each data source characteristics on this separability. Assessments have shown it is hard to distinguish individuals based only on their walking patterns in a subject-based identification scenario. In this setup, the subjects’ separability is more related to their physical characteristics than their movements related to gait cycles and biomechanical events. However, this study’s results also points to the feasibility of learning identity characteristics from individuals’ walking patterns learned from similarities and differences between subjects in a verification setup. The explorations concluded that periodic components occurring in frequencies between 6 and 10 Hz are more significant for learning these patterns than events and other biomechanical movements related to the gait cycle, as usually explored in the literature.

## Introduction

*Walking* is defined as “a method of locomotion involving the use of the two legs, alternately, to provide both support and propulsion”^1^. Gait differs from walking because the latter is related to the process itself, whereas gait describes “the manner or style of walking”^1^. Particularities in the manner of walking have been explored for at least three decades as a biometric trait able to discriminate individuals^2^. The first explored modality was a video-based analysis by generating contours from images collected by fixed cameras and extracting spatiotemporal gait patterns from these contours^3^. Three years later, the modality of sensor floors was proposed, involving a weight-sensitive floor with sensors to measure vertical forces and using them to construct footstep signatures of the individuals^4^. The most recent modality of gait recognition is based on inertial sensor data, such as features extracted from accelerometer data being captured using portable devices^5^. This last one, inertial-sensor-based gait biometrics, has been increasingly explored to prevent unauthorized use of personal devices, especially as mobile and wearable devices with embedded inertial sensors have become more popular and resourceful^6–10^. This can become a powerful security layer, as the manner of walking is hard to spoof and could identify users passively and transparently, that is, without users being required to perform any action^7,11^.

Although biomechanical aspects are extensively exploited in gait analysis literature^12^, gait recognition works usually do not discuss which factors of gait traits are essential to distinguish individuals^7,11,12^. Cycle-based approaches rely on detecting gait cycles from inertial data to find a gait pattern for each subject, and these gait patterns are used as templates in a direct matching or as input to a feature extraction process. Frame-based approaches segment inertial data into fixed-length frames and usually rely on extracting generic features from these frames. These features usually are based on the computation of simple parameters, such as cycle length and gait cycle frequency, when analyzing gait cycle; or frequency-domain coefficients like Fast Fourier Transform (FFT) and Wavelet coefficients; or, statistical parameters (e.g., mean, standard deviation, skewness, kurtosis) and histograms^13^. Typically, the criteria for choosing specific features and performance measures are not commonly related to or inspired by biomechanical factors and gait analysis theory^7,11,14,15^.

Furthermore, the recent adoption of deep learning techniques in inertial-sensor-based gait recognition approaches has been adding more uncertainty about the traces being learned to recognize or identify subjects. In these approaches, deep learning architectures (e.g., Convolutional Neural Networks (CNN) and Recurrent Neural Networks (RNN)) learn features directly from data while minimizing the model error^16–19^. Features extracted by these architectures are not easily understandable, and could be more related to specific dataset characteristics, or biases, rather than to general gait traits^20^.

Consequently, results from current research in gait biometrics have seldom been contrasted to insights from the biometrics domain, which means that the prediction accuracies in particular datasets are the main evidence that current systems for gait biometrics can generalize to a production setting. However, the datasets used in most applications lack a controlled environment for proper analysis, thus improved results could be due to the exploitation of myriad of confounding factors such as shoes, clothing, and walking surfaces^21–23^. This disconnection between domain knowledge and the approaches used in biometrics, that is, the lack of interpretability, harms both the reliability of current methods and the development of new ideas for this problem. Our work aims to fill this gap bringing to bear important factors that must be considered when designing a biometrics system and aspects that should be accounted for. In other words, it is not sufficient to look at the performance figures; it is necessary also to understand the factors driving (up or down) such figures and our work aids this analysis.

This work addresses this interpretability problem by directly investigating which gait traces make individuals distinguishable from others. These traces are assessed both in user identification and user verification scenarios. The assessments were designed to answer questions about the reliability and generalization of walking patterns as identity characteristics capable of distinguishing individuals. This study presents evidence toward understanding whether gait cycle events and biomechanical movements, usually considered in gait analysis, are helpful discriminative components in gait recognition and identification applications.

For this study, a multi-sensor gait dataset was used to investigate the impact of each sensor’s data acquisition characteristics on subject identification. This dataset comprises synchronized inertial and optical motion data and was captured under controlled conditions, allowing the comparison of gait analysis aspects from both systems without the interference of external factors such as differences in the floor, walking paths, or shoes. Optical motion data provides three-dimensional trajectories more precisely but requires specific installations in a controlled environment. The inertial sensor is a low-cost device that captures accelerations unobtrusively but is simultaneously more susceptible to capture noise. The two sources were used to identify which gait traces generalize along with both.

The separability patterns were assessed using manifold projection techniques. These analyses were carried out in light of gait and biomechanics theory. They aim at explaining the relation of walking movement components to gait cycle events and other biomechanical movements occurring within the gait cycle. This study is also helpful in determining essential factors for developing more reliable features in a gait recognition approach.

## Results

This section presents questions about gait acting as a biometrics modality and assessments aimed to answer them. All investigations were carried out using the dataset described in the Methods section. The analyses used the generated frames of marker trajectories and the microcontroller’s (MCU) accelerometer readings.

**Figure 1:**
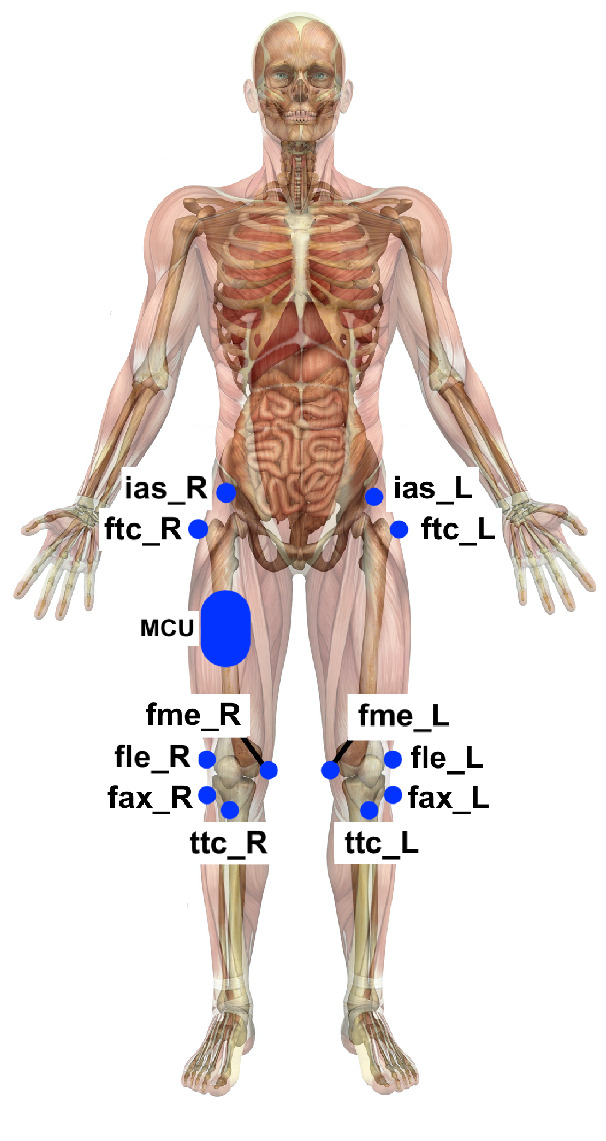
Landmarks of the analyzed markers near the MCU location. Drawn by the authors. ias: Anterior-superior iliac spine; ftc: Most lateral prominence of the right greater trochanter; fme: Most medial prominence of the medial femoral epicondyle; tcc: Most anterior border of the tibial tuberosity; fax: Proximal tip of the head of the fibula; fle: Most lateral prominence of the lateral femoral epicondyle; MCU: Microcontroled device with the accelerometer. In Fig. 1, the MCU is placed on the left thigh, but it was exchanged between both legs, as described in^24^.

Because the MCU accelerometer was placed on the middle of the thigh, the markers located on landmarks near it were used as reference. These landmarks are listed bellow, and their placement jointly to MCU location are depicted in Fig. 1.

This work adopts the Uniform Manifold Approximation and Projection for Dimension Reduction (UMAP) to perform the visualization and separability analysis. UMAP aims to construct a topological representation of the high-dimensional data and use it to optimize its equivalent low-dimensional topological representation using local fuzzy simplicial sets^25^. This implies a low-dimensional data projection that is highly correlated to the original high-dimensional representation.

### How to model the walking movement?

Walking movements are captured by sampling a continuous-time signal at regular time intervals *T*. This process results in a discrete-time representation *x*[*n*] of the continuous signal *X*(*t*) given by: $x[n] = X(nT)$, in which *n* is the sample number. The sampling rate $F_s$ defines the number of samples per second, and is given by $F_s=1/T$^26^.

The walking movement is composed by gait cycles. A gait cycle comprehends the time interval between the two consecutive occurrences of one of the walking events^1^. These cycles comprise two phases, **stance** and **swing**, and particular walking events, such as toe off and initial contact. As gait is composed of several repetitions of walking events, Fourier Series can be a valuable approach to describe it.

The Fourier Series models periodic signals by weighted sums of complex exponentials whose frequencies are integer multiples of a known fundamental frequency *F*^26^. This process allows to observe a great energy concentration in frequencies that are multiples of *F*. The Discrete Fourier Transform (DFT) is commonly employed to obtain the Fourier representation of a discrete signal. The DFT of a finite-length sequence relates to its Discrete Fourier Series by highlighting the energy concentrations in the frequencies that are multiples of the fundamental frequency, which appear as lobes in the signal’s DFT coefficients. The DFT of a discrete signal is defined in Eq. (1).

1 $$\begin{aligned} X[k] = \sum ^{N-1}_{n=0} x[n] e^{-i \frac{2\pi k}{N} n} \end{aligned}$$

The DFT yields a frequency-domain signal *X*[*k*], where *X*[*k*] is the signal’s energy at frequency $k F_s/N$ Hz. In this case, *N* is the window length used to calculate the DFT. In the DFT coefficients *X*[*k*] appear lobes of higher magnitude when $k F_s/N$ Hz corresponds to a frequency multiple of the fundamental *F*. However these coefficients highlights frequency components that are not necessarily part of the Fourier Series of *x*[*n*].

**Figure 2:**
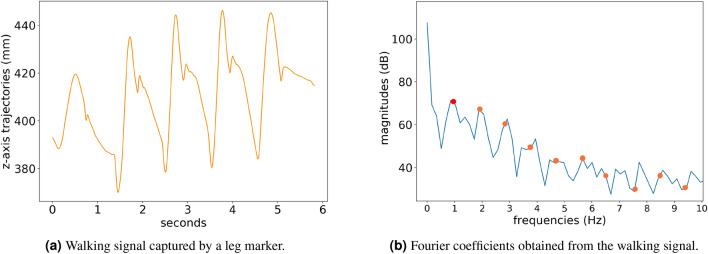
An example of walking signal captured by a leg marker (2a), and a zoom from 0 to 10 Hz of its corresponding Fourier coefficients (2b) with the fundamental frequency (circle in red) and the other harmonics (circles in orange) highlighted.

An example of a frequency-domain signal *X*[*k*] obtained from marker trajectories is shown in Fig. 2. This figure presents a walking signal obtained using a leg marker on the left side, and zoom from 0 to 10 Hz of the DFT coefficient magnitudes obtained from this signal, expressed in units of decibel (dB), on the right side.

As presented in Fig. 2, the magnitude of frequency 0Hz (the DC component) has a notably high magnitude. This component represents the signal’s average value. Also, this figure depicts that the signal’s fundamental frequency is around 0.94Hz (circled in red), thus its harmonics (represented by orange circles) are at$$\begin{array}{cccccc}\{0.94Hz&1.88Hz&2.82Hz&3.76Hz&\dots&m*0.94Hz\}, \end{array}$$ in which *m* is the *m*-th harmonics. As discussed before, the harmonics are associated with lobes of higher magnitude, which can be seen as local maxima in the DFT magnitudes.

There are also information represented by the energy accumulated in non-harmonics frequencies. For example, important periodic information are located on frequencies between the harmonics, called intra-harmonics. In the case of walking signals, the DFT highlights: (i) relevant gait characteristics related to the harmonics (e.g., gait cycles, steps); (ii) the DC component that describes the data bias; and (iii) important periodic information unrelated to the gait cycle in the non-harmonic frequencies. Therefore, the DFT coefficients were adopted as feature vectors for most analyses of this study.

#### How reliable is it to differentiate subjects based on their gait using their trajectories or accelerations?

**Figure 3:**
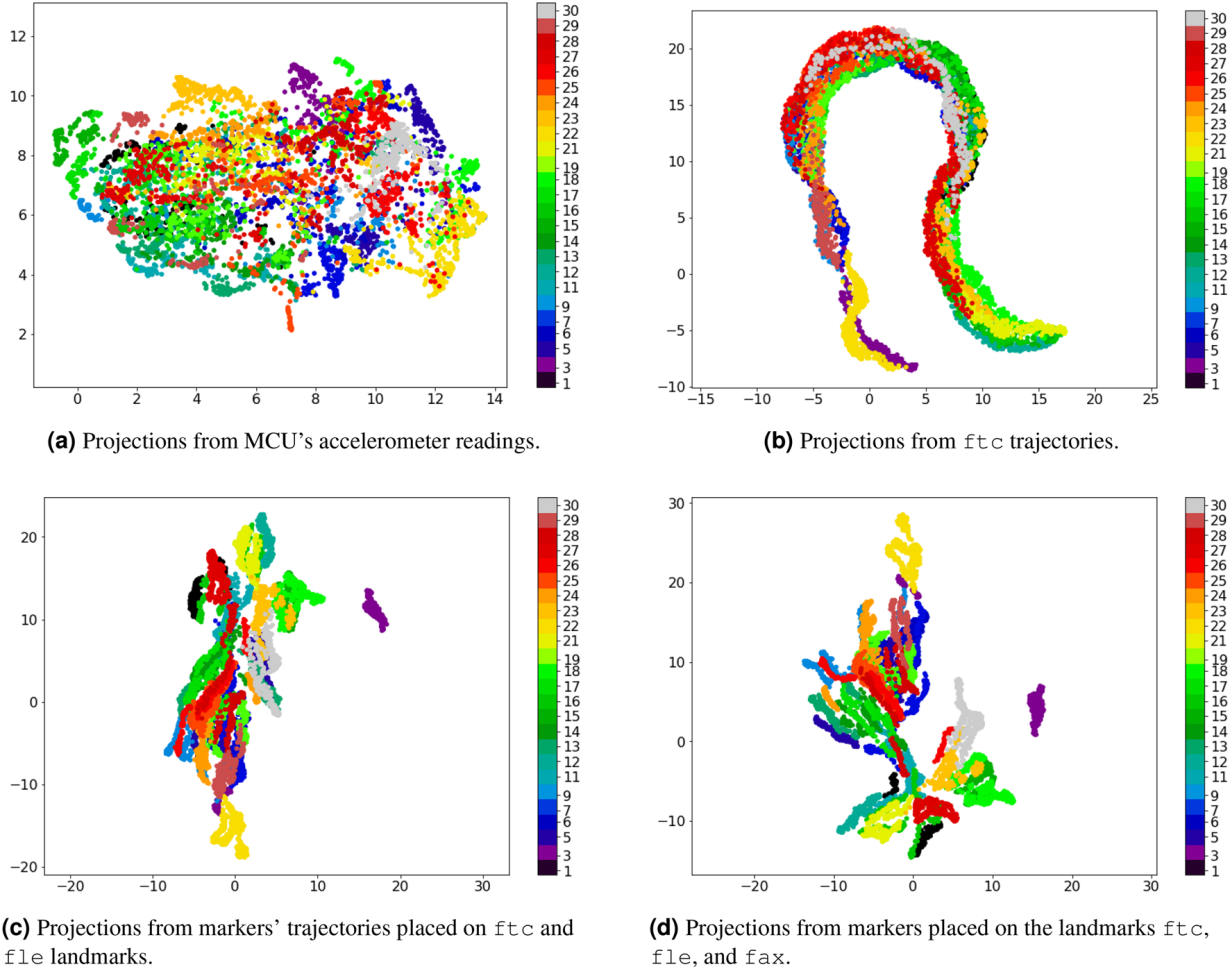
Bi-dimensional UMAP projections using the DFT coefficients of different signal sources. Each color corresponds to a subject. The MCU accelerometer readings lead to more spread clusters than the trajectories. Using more trajectory markers lead to more compact clusters.

Figure 3 presents the scatter plot of the UMAP bi-dimensional projections using feature vectors extracted from MCU’s accelerometer readings, and trajectories of the most relevant analyzed markers. Because of the DFT symmetry property, these features were extracted for each frame by adopting the DFT coefficient magnitudes of frequencies higher and equal to 0 Hz. The resulting projections were plotted using a scatter plot, in which the colors differ from the 25 subjects of the dataset according to the color bar on the left side of each plot. This clustering of subjects relates to how gait recognition based on subject identification works, which aims to achieve separability between the dataset subjects.

The subjects’ clusters generated by the accelerations’ projection (Fig. 3a) are weakly separated, and only a few of these clusters are reasonably defined, such as the individuals of id 5, 23, and 30. Likewise, the projections calculated from only one marker’s trajectories (Fig. 3b) do not provide a clear cluster visualization. However, the clusters from trajectories still visually less spread than in Fig. 3a.

Furthermore, concatenating feature vectors from more than one marker simultaneously (Fig. 3c,d), leads to clusters gradually more delineated, and the distances among these clusters become larger. However, this analysis is still inconclusive about whether this difference is due to the nature of the measured phenomenon (trajectory vs. acceleration) or to possible additional identity information captured by trajectories.

#### Is there any additional subjects’ identity information about the subjects in accelerometer’s and markers’ trajectories besides the way they walk?

**Figure 4:**
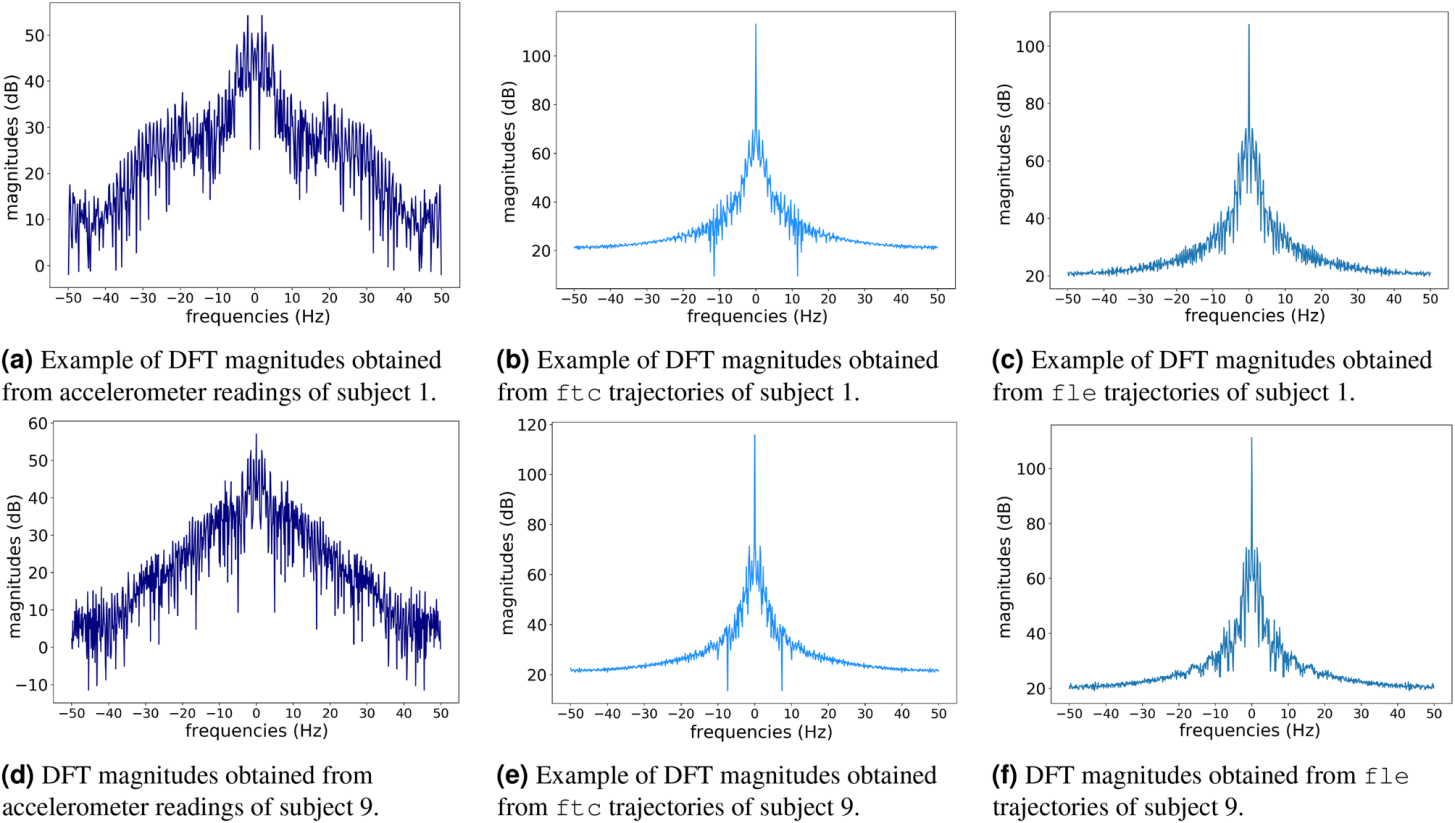
Examples of DFT magnitudes (in units of decibel) extracted from markers’ trajectories and MCU’s accelerometer readings captured by two random subjects. First row shows the DFT magnitudes ftc and fle trajectories, and accelerations of a subject-one’s trial. The second row presents the corresponding DFT magnitudes of a subject-nine’s trial.

According to Fig. 3 and its previous discussion, trajectories captured by markers on body landmarks might comprehend identity information additional to gait traits. This hypothesis can be verified by first investigating the Fourier coefficients of the accelerations and trajectories, aiming to notice any difference interpreted as identity information. Figure 4 presents DFT magnitudes (in units of a decibel) calculated from markers trajectories (fle and ftc) and MCU’s accelerations of two different random subjects.

**Figure 5:**
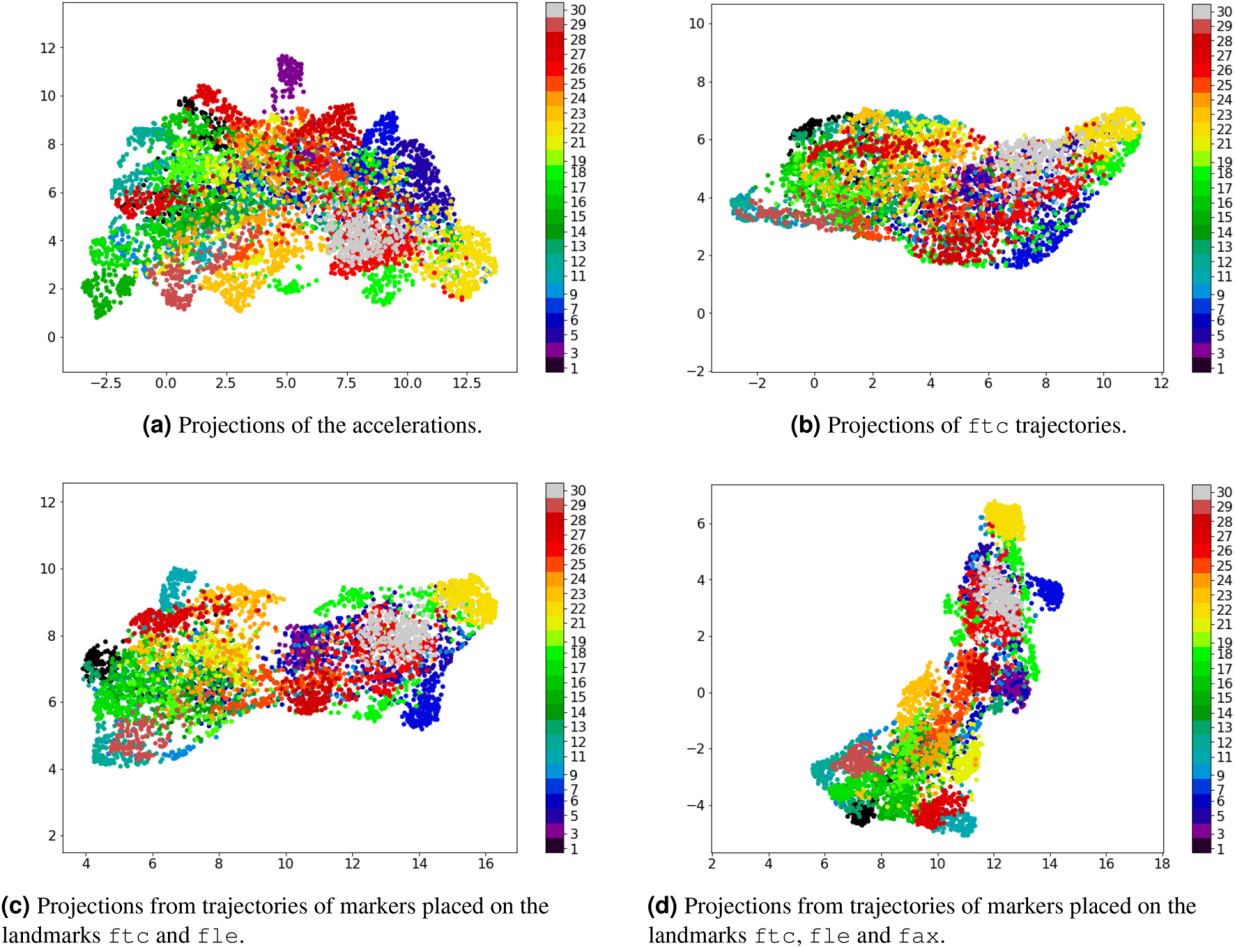
Bi-dimensional UMAP projections using the DFT coefficients of different signal sources after being stop-band filtered at 0Hz. Each color corresponds to a subject. After removing DC component, the trajectory clusters become more spread. However, this almost not affect the MCU accelerometer readings because its DC component corresponds to the gravity.

According to the examples in Fig. 4, the DC components (magnitude in 0 Hz) of both markers are higher than the magnitudes of other frequencies for both subjects. This does not occur to DFT magnitudes from the accelerations. It suggests more impact of trajectories’ bias than the accelerations’ one on the learned identity characteristics, which might explain the differences among the projections of Fig. 1. The DC component was removed from all trajectories and accelerations by a band stop filter at 0 Hz to verify this hypothesis. Figure 5 shows the projections after band-stop filtering of the markers’ trajectories and accelerometer readings.

The DC component on the DFT magnitudes of trajectories describes identity characteristics related to the subject’s height, as it is related to their body parts’ lengths. For example, the fle marker is placed on the most lateral prominence of the lateral femoral epicondyle, which is approximately the subject’s knee height. Figure 5b,d demonstrate the impact of DC component on the trajectory feature vectors, reducing the subject separability when removing it (in comparison to Fig. 3b,d. Thus, the trajectories carry identity information unrelated to gait traits; this information is more permanent (between different days) and has a higher impact on the subject’s identification process than the gait itself. Importantly, this aspect is also present on vision-based gait recognition, in which most works use silhouette extraction or pose estimation to generate the features^2^.

In turn, the DC component on the DFT magnitudes of the accelerations represents the environment gravity force. This is the same for all the dataset subjects, once they captured data on the same straight-level walkway and controlled environment. Figure 5a confirms this, depicting a similar separability among subjects to the one shown in Fig. 3a. Therefore, in this case, the DC component does not contribute to the subject’s identification, which explains the differences among the resulting projections showed in Fig. 1.

#### How reliable and generalizable is it to learn common characteristics intra- and inter-individuals?

Based on the previous discussion about additional identity information acquired by the subject’s trajectories, further analyses are performed using trajectories and accelerations filtered by a band-stop filter at 0 Hz (to remove the DC component).

Figure 5 establishes a specific limitation of subject identification using biometrics information without the subjects’ body characteristics. However, another approach commonly adopted in biometrics recognition is subject identity verification. In this problem, there is only one subject of interest (the authorized subject, usually the device owner), and data is used to learn the authorized subject patterns in contrast to patterns of non-authorized subjects.

This verification process can be performed by analyzing the relationships between pairs of feature vectors, aiming to verify whether they are from the same subject or from different individuals. This leads to a type of model that relies on the hypothesis of being viable to learn separability patterns through analyzing pairs of feature vectors from same subject and from different subjects.

This methodology was applied in Santos et al.^8^, in which a deep-learning-based solution was proposed to learn profiling patterns of “same user” and “different users”, and achieve a user-agnostic method for identity verification based on motion traits. Applying this idea, feature vectors composed by DFT components of the individuals were combined. In this case, pairs of feature vectors from same and different subjects were subtracted and element-wise multiplied. Using a mathematical notation, these same-subject pairs using the *i*-th and *j*-th feature vectors, *fv*, of the subject $\gamma$ were combined by:

$$\begin{aligned} (|fv_{\gamma _i} - fv_{\gamma _j}|, |fv_{\gamma _i} \odot fv_{\gamma _j}), \end{aligned}$$

in which $1 \ge x \le 25$, $1 \ge i \le N_\gamma$ and $1 \ge j \le N_\gamma$ being $N_\gamma$ the total number of frames of the subject $\gamma$.

Whereas, different-subjects pairs using the *i*-th and *k*-th feature vectors of subjects $\gamma$ and $\zeta$, respectively, were combined by:

$$\begin{aligned} (|fv_{\gamma _i} - fv_{\zeta _k}|, |fv_{\gamma _i} \odot fv_{\zeta _k}), \end{aligned}$$

in which $1 \ge x \le 25$, $1 \ge y \le 25$, $x \ne y$, $1 \ge i \le N_\gamma$ being $N_\gamma$ the total number of frames of subject $\gamma$, $1 \ge k \le N_\zeta$ being $N_\zeta$ the total number of frames of subject $\zeta$.

**Figure 6:**
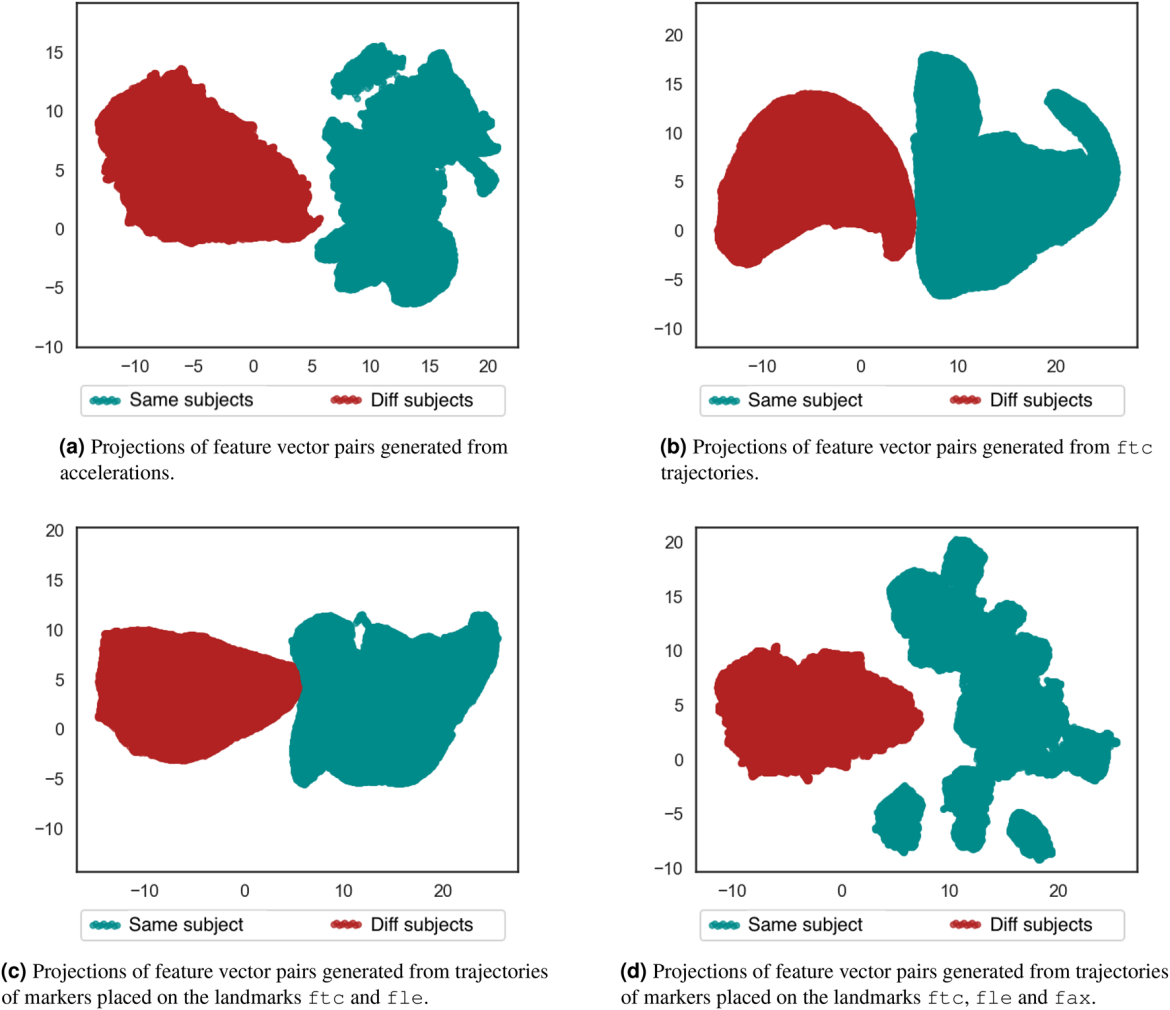
Bi-dimensional UMAP projections using the feature vector pairs generated from different signal sources without the DC component. The clusters of pairs from same subject and different subjects are visually separated. Combination of trajectories from additional markers decreases the separability.

Figure 6 depicts the UMAP bi-dimensional projections produced by UMAP using the pairs of same and different subjects, which were created through the DFT feature vectors from the subjects’ trajectories and accelerations.

As shown in Fig. 6, learning separability patterns between pairs of feature vectors from same and different subjects is more viable than discriminating individuals by their gait characteristics. An interesting fact when comparing Figures 5 to 6 is that additional markers improve the subjects’ identification, but make the learning of relationships from the pairs more complicated, specifically for the pairs from the same subject. This can be related to the difficulty of finding common characteristics given the variability increasing related to specific components of each marker trajectories (e.g., noise generated by the marker movement on the skin, more variability on marker placement, noise related to the calibration errors and occlusion). This indicates that using a single marker is enough for subject verification.

**Figure 7:**
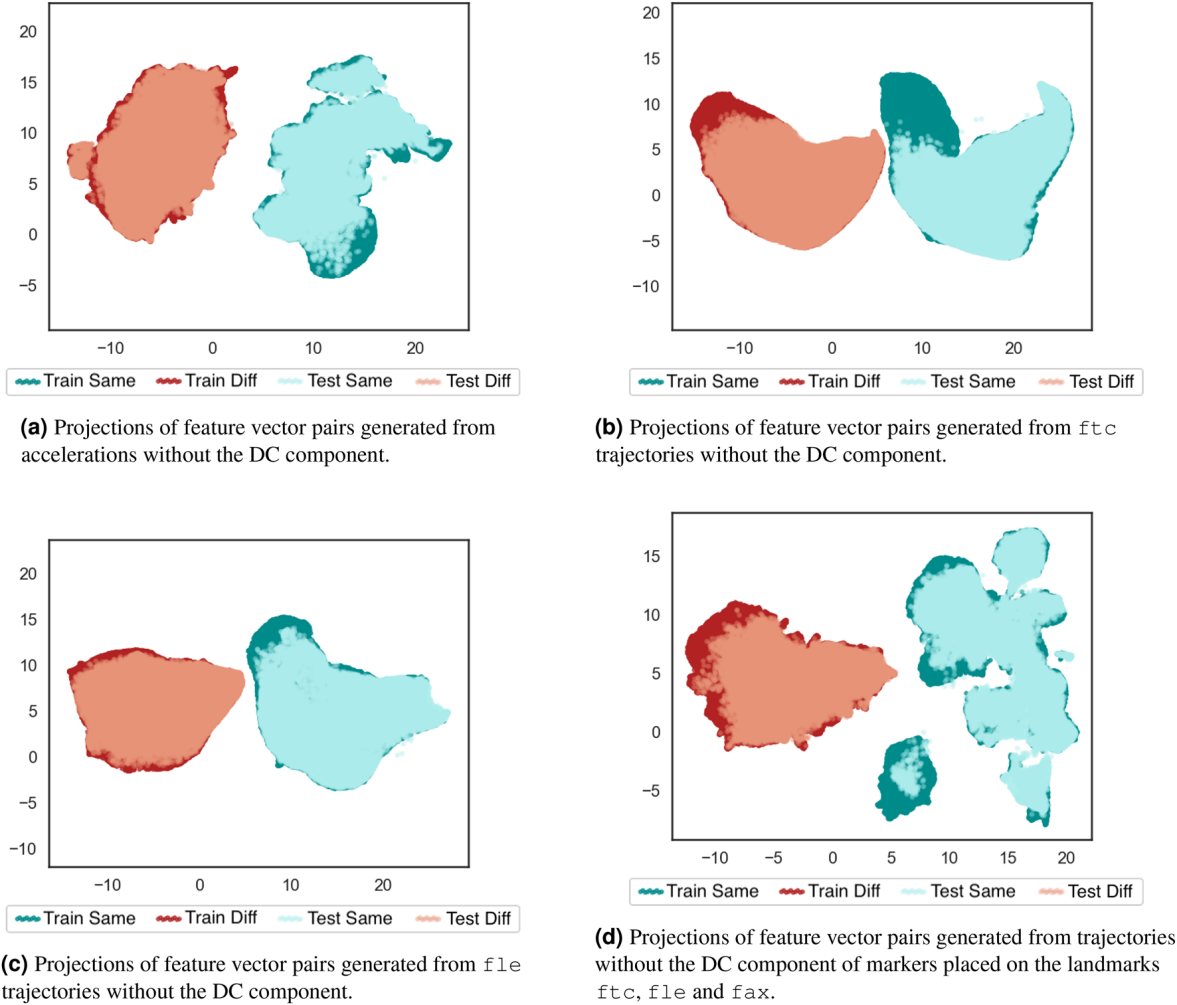
Bi-dimensional UMAP projections using the feature vector pairs generated from different sources after removing the signals’ DC component. Four folds of pairs were generated: training set of same-subject pairs, training set of different-subjects pairs, test set of same-subject pairs, test-set of different-subjects pairs. The clusters of pairs from same subject and different subjects are visually separated for training set and test set. The overlap between training set and test set pairs indicates generalization between different set of individuals.

A concern about learning the separability patterns from same-subject and different-subjects pairs refers to their generalization capability when adding data from new individuals. This was assessed by training a supervised UMAP using data from half of the dataset subjects (training subset) and validating the learned patterns using the remaining 12 individuals’ data (test subset). Same-subject pairs kept being generated using each subject’s feature vectors from acquisition of first and second day, whereas different-subject pairs were obtained combining only feature vectors from individuals of the corresponding subset, training or test. Figure 7 shows the obtained bi-dimensional projections from these same-subject and different-subjects pairs using supervised UMAP. The resulting projections of pairs generated using feature vectors from individuals of training and test sets are plotted to verify the generalization achieved by the learned separability patterns.

By comparing plots of Fig. 7 to the ones of Fig. 6, it is noticeable that learning relationships between feature vector pairs of same and different subjects using supervised UMAP allowed to improve the clusters separability and generalize this to testing subjects. Figure 7d follows the previous pattern of Fig. 6d, in which the variability increasing of additional marker feature vectors interferes on the learning of those relationships, specially for the same-subject pairs. Considering this factor and the generalization aspect, further experiments will be presented using supervised UMAP and feature vector pairs from one marker trajectories. The adopted marker is the fle, because it presented better separability patterns in supervised UMAP than the ftc. Investigations using MCU’s accelerometer readings are also important to compare trajectory patterns to acceleration ones. Supplementary Figs. S1–S3 provide investigations about training UMAP using other random individuals’ subsets and reducing the number of subjects for training to three and six. This allows assessing generalization aspects of the learned similarities and dissimilarities using other possible combinations of subjects for the training, and the impact of reducing the number of training individuals to achieve this generalization.

#### What information is more relevant to determine identity characteristics present in walking data?

**Figure 8:**
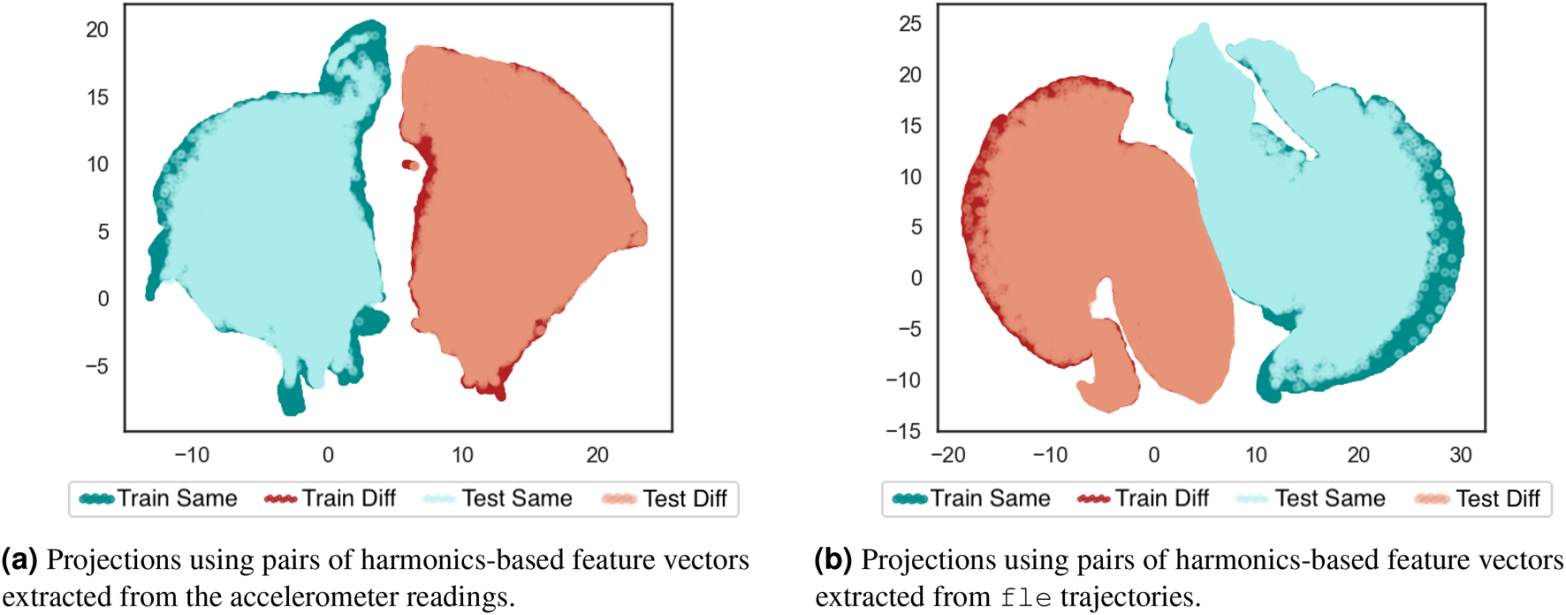
Bi-dimensional UMAP projections using the pairs of harmonics-based feature vectors generated from different signal sources. Four folds of pairs were generated: training set of same-subject pairs, training set of different-subjects pairs, test set of same-subject pairs, test-set of different-subjects pairs. The clusters of pairs from same subject and different subjects are weakly separated.

In the Fourier Series model for gait, the fundamental frequency refers to the gait cycle frequency, whereas other events which subdivide the gait cycle and have common movements (e.g., flexion, extension, changes between flexion and extension) encoded in the magnitudes of the harmonics. This property leads to feature vectors consisting of the fundamental frequency and the magnitudes of the first 10 harmonics. From these feature vectors, pairs from the same and different subjects were constructed as described previously. Figure 8a,b show the resulting two-dimensional UMAP projections of these harmonics pairs calculated from MCU’s accelerometer readings and fle marker trajectories, respectively.

According to Fig. 8, harmonics information might not be enough to provide a good separability between feature vector pairs of same and different subjects. Particularly, when these projections are compared to those from DFT features using all frequency components (without the DC component) in Fig. 6, the clusters obtained from the harmonics pairs are poorly delineated and closer to each other. As this harmonics analysis demonstrated inadequate to provide better distinctness between pairs of same and different individuals, investigations about frequency ranges were designed.

**Figure 9:**
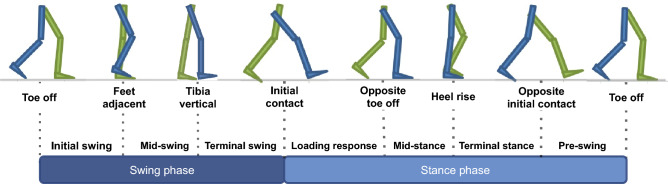
Phases of a gait cycle and the positions of legs during walking events of a cycle started by the right leg (in blue). Drawn by the authors, based on BoH, File Walk cycle, CC BY-SA 4.0, via Wikimedia Commons.

**Figure 10:**
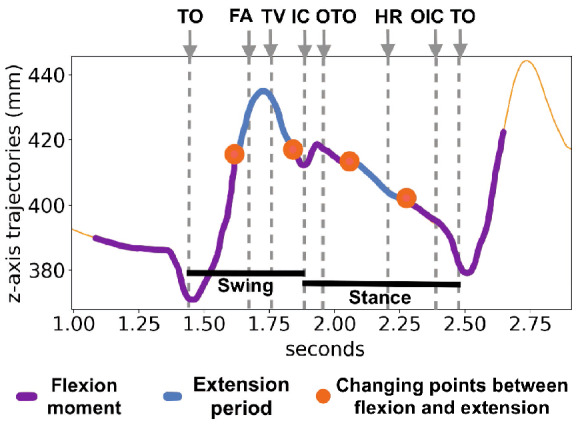
Gait cycle events observed on fle marker trajectories, including the moments of flexion and extension. TO: toe off; FA: feet adjacent; TV: tibia vertical; IC: initial contact; OTO: opposite toe off; HR: heel rise; OIC: opposite initial contact.

Given that individuals usually walk at a maximum stepping frequency of about 2.5 Hz^27^, their maximum cycle frequency is around 1.25 Hz. Gait events were reconstructed through the 3D skeleton view of QTM software. Considering that fle presented better pairs discrimination among the analyzed markers and it is a landmark of the knee, the gait cycle reconstruction was based on the knee movements. The QTM skeleton view in conjunction with fle landmark trajectories, as well as knee joint angle reference^1^, were used to approximately determine the moments of gait events in this landmark. Figure 9 shows the gait events, phases and the corresponding feet positions of a gait cycle by the right leg. These events and phases, as well as the periods of flexion and extension, and the approximated moments which happen changes between flexion and extension for the fle landmark trajectories are depicted in Fig. 10. The comparison between these two Figures allow to understand the movements performed by the knee during a gait cycle.

**Figure 11:**
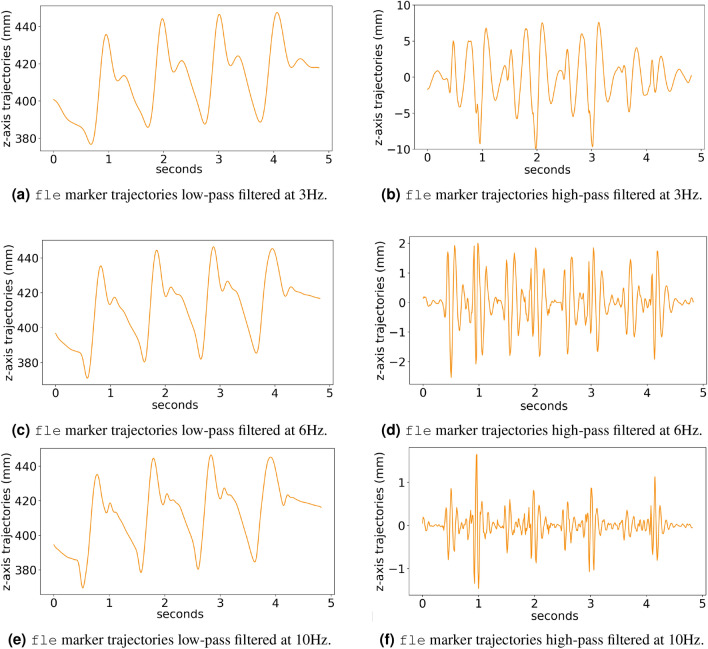
Example of fle trajectories filtered by low-pass and high-pass filters at 3Hz, 6Hz and 10Hz.

The knee presents two flexion and two extension peaks during each gait cycle^1^. Thus flexion periods, as well as extension ones, occur twice in a cycle. The points in which flexion and extension are exchanged appear four times in a gait cycle, i.e., double stepping frequency. Therefore, all gait events and movements of fle landmark might occur at a maximum frequency of four times the maximum cycle frequency, i.e., about 5 Hz. Figure 11 shows examples of fle trajectories low-pass and high-pass filtered at three different frequencies: 3 Hz, 6 Hz and 10 Hz. A low pass with a cutoff frequency of 3 Hz guarantees the inclusion of frequency components related to gait cycle, gait events, and steps. A cutoff frequency of 6 Hz encompasses other possible movements within a gait cycle, such as the changes between flexion and extension. The cutoff frequency of 10 Hz was chosen because, higher than this frequency, the DFT magnitudes are drastically reduced compared to magnitudes of the lower frequencies.

Comparing Figs. 10 to 11a, some movements related to gait cycle are not present in the 0 Hz–3 Hz band of the fle trajectories. Additionally, whereas z-axis fle trajectories of a complete gait cycle, in this example, show a displacement of about 60 mm, fle trajectories are high-pass filtered at 3 Hz yet demonstrate a displacement of about 20 mm. This represents one-third of the main gait cycle displacement and confirms that movements within a cycle are not completely described in frequency components up to 3 Hz. On the other hand, by comparing Figs. 10 to 11c, the gait events and related biomechanical movements are represented in this fle trajectories low-pass filtered at 6 Hz. This fact is ascertained by the low range displacement of z-axis fle trajectories high-pass filtered at 6 Hz in Fig. 11d, only about 4 mm. The trajectories low-pass filtered at 10 Hz, in Fig. 11e, are similar to the ones low-pass filtered at 6 Hz (Fig. 11c), reassuring that no relevant periodic related to the gait cycle occur in a frequency higher than 6 Hz. Figure 11f also demonstrates an even lower range displacement of the fle trajectories high-pass filtered at 10 Hz—of about 2 mm, which is closer to the average residuals and standard deviation found in the system calibration^24^.

**Figure 12:**
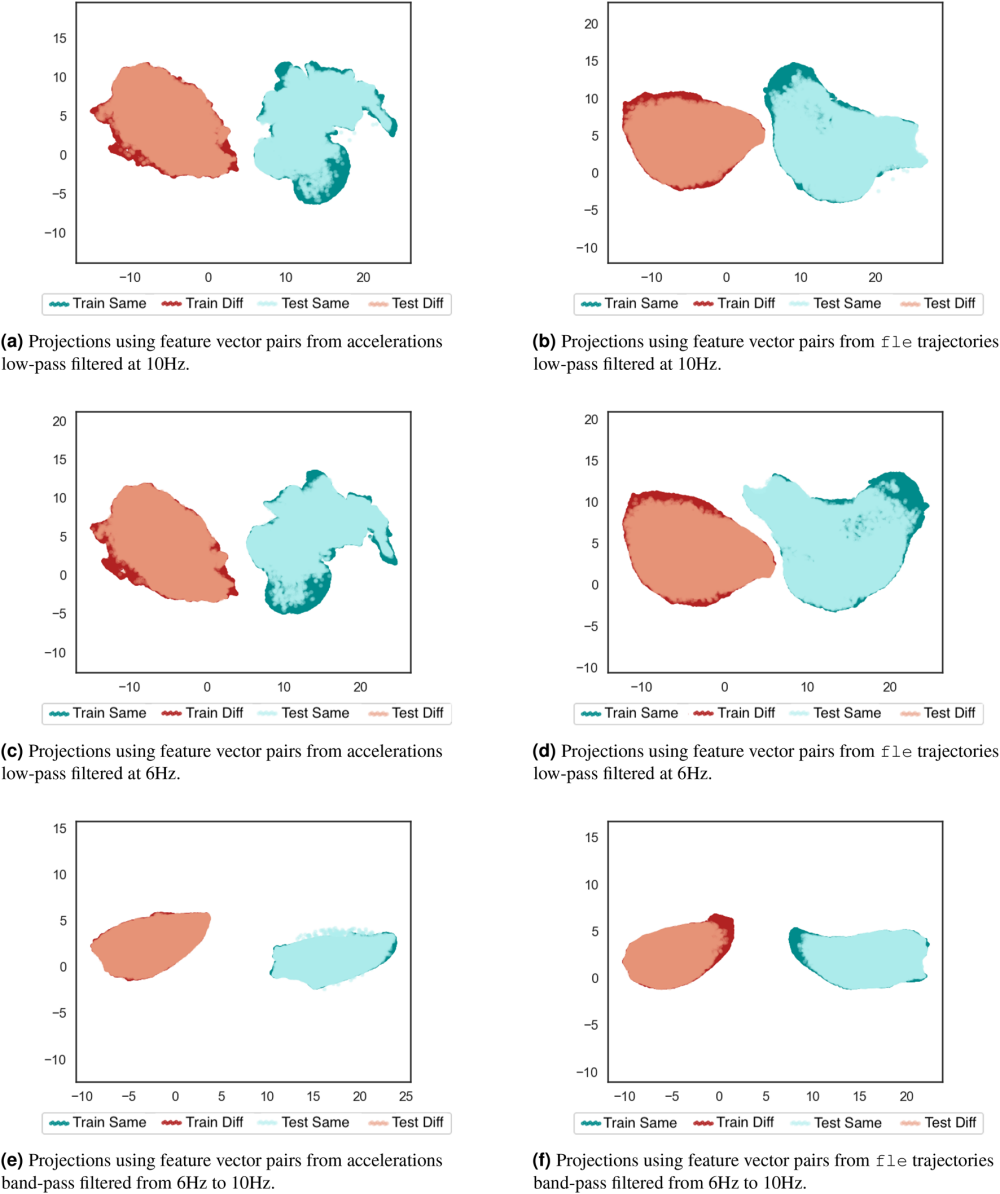
Bi-dimensional UMAP projections using the feature vector pairs generated from accelerations and marker trajectories filtered at different frequencies. Four folds of pairs were generated: training set of same-subject pairs, training set of different-subjects pairs, test set of same-subject pairs, test-set of different-subjects pairs. The band-pass filtering from 6 to 10 Hz lead to the greatest separability for the pairs. The train and test sets consistently overlap, indicating a robust learned representation.

Based on these observations of Fig. 11, 2-D UMAP projections were generated from DFT components of MCU’s accelerometer readings and fle trajectories low-pass filtered at 10 Hz, and 6 Hz. In addition, the trajectories and accelerations were band-pass filtered using the cutoff frequencies 6 Hz and 10 Hz. This band-pass filter is useful to verify the achievable separability using periodic information not properly related to events and biomechanical movements of the gait cycle but removing possible noises and system-related information comprised in frequencies higher than 10 Hz. This manifold projection experiment adopted the same process of training UMAP with feature vector pairs of same and different subjects, using a subset of 13 individuals, and validating with pairs of the remaining 12 individuals. The resulting 2-D projections are depicted in Fig. 12.

The impact of removing components of frequencies higher than 10 Hz and those higher than 6 Hz was not expressive to improve the separability between feature vector pairs of same and different subjects, when comparing Figs. 12b,d to 7c, and Figs. 12a,c to 7a. This confirms that magnitudes of frequencies lower than 10 Hz are considerably higher than those of frequencies higher than 10 Hz, and most gait-cycle-related movements are described in frequencies lower than 6 Hz. However, the projections using feature vector pairs of accelerations and fle trajectories band-pass filtered from 6 to 10 Hz (Fig. 12e,f) demonstrated better separability between those pairs of same and different individuals than those obtained using accelerations and trajectories low-pass filtered at 6 Hz and at 10 Hz, as well as better generalization observed by applying the learned separability patterns from training subjects to testing ones. Also, the clusters generated using the pairs from accelerations, Fig. 12e, present similar uniform distribution to those achieved from fle trajectories, Fig. 12f, which indicates similar information being captured by trajectories and accelerations within this frequency range. This analysis suggests that the motion information able to distinguish identity characteristics of trajectories and accelerations from same and different subjects are not exactly correlated to the events and biomechanical movements related to the gait cycle.

Although the frequency components between 6 and 10 Hz are still related to periodic movements, the associated movements to this frequency range are not fully explained in gait analysis literature. Usually, a 6 Hz cutoff frequency is used to perform kinetics and kinematics analysis^28^. Hence about 99% of the signal power is concentrated up to this frequency^29^. However, some recent works have adopted a cutoff frequency of 10 Hz to cover additional biomechanics’ applications range^28^. Some examples of relevant components present in frequencies above 6 Hz are ground stabilization, impact forces—both related to the body mass^30^; and muscles vibration, associated with the individuals’ muscle fiber compositions^31^.

**Figure 13:**
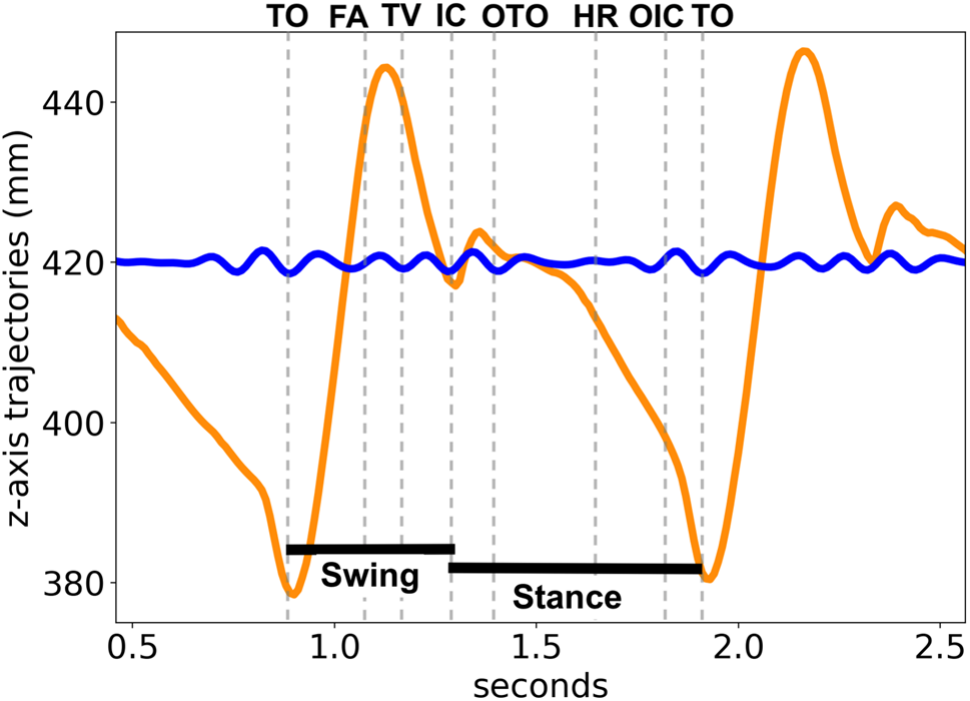
A gait cycle of raw fle marker trajectories and of these band-pass filtered from 6 to 10 Hz. The gait events are defined and highlighted as in Fig. 10.

Figure 13 depicts the gait events highlighted in raw trajectories captured by the fle marker, and corresponding trajectories band-pass filtered from 6 to 10 Hz, being possible to determine the approximated events in which these high-frequency components (between 6 and 10 Hz) are correlated. The magnitudes of these components are visually higher in the swing phase than in instance one. A similar pattern of these components occurs near the intervals between initial contact and opposite toe-off and between opposite initial contact and toe-off. This coincides with vertical ground forces found in the double-hump pattern occurring near opposite toe-off and before opposite initial contact^30^. Additionally, experiments using electromyography sensors (EMG) demonstrated thigh muscle excitation patterns between initial contact and opposite toe-off, between opposite initial contact and toe-off, and before initial contact^32^. *These components demonstrated to be more relevant as identity characteristics to distinguish individuals than events and movements related to the gait cycle*.

## Discussion

The investigations have shown the difficulty of distinguishing individuals based on their walking trajectories or accelerations in a subject identification scenario, even in a dataset captured under controlled conditions. Additionally, this study demonstrates that subjects’ identification, usually employed in gait recognition literature^12,16^, is more related to these subjects’ physical characteristics, such as height or length of body components, than their movements related to gait cycles and events.

However, the results of this study also pointed the viability of learning identity characteristics from individuals’ walking trajectories or accelerations through separability patterns obtained according to similarities and differences among *feature vector pairs* of same and different subjects. Furthermore, these patterns presented generalization capability by learning them from a subset of individuals and extending it to another subset containing unknown ones.

The identity characteristics were explored to comprehend which individuals’ traces are more relevant to improve the separability patterns. The explorations concluded that periodic components occurring in frequencies between 6 and 10 Hz are more significant for learning these characteristics than events and other biomechanical movements related to the gait cycle. These components are not entirely explained in gait analysis literature, although it is conceivable to state the relation of this frequency range to muscle excitation and vertical ground forces patterns^30,32^, for example.

These results can be used to direct the development and analysis of human identification systems based on the way of walking—gait. More importantly, they indicate traits that are more likely to contain discriminating features, which can help avoid confounding factors or coincidental, dataset-specific features (like variations due to clothes, shoes, or terrain) by the purposeful design of user identification systems and result in analysis specifically focused on finding these elements. This could cause identification systems to account for (and potentially limit) data-specific characteristics present in particular datasets, leading to worse performance in particular benchmark datasets, even if their generalization in production settings is likely to improve, which is ultimately the desired system. Also, the analysis of such factors is also important to direct debiasing algorithms, i.e., algorithms to help account for confounding (bias) factors in the datasets.

These findings were possible because multimodal data acquisition allowed separating user-specific from the sensor-specific characteristics. This type of data acquisition is hard to perform outside a laboratory environment, in special due to the optical motion sensors. This has reduced the potential interference of external factors during the analysis, henceforth elements such as clothing, shoes, and terrain could be accounted for. .

A potential extension of this study is to verify the suitability of the found conclusions using in-the-wild data captured in a non-controlled environment, and considering daily-life conditions with users wearing/using sensors all-day long. Such study could gather more evidence indicating the links between motion signal characteristics and other aspects not directly linked to the users, like the terrain or the clothing.

Although subjects had diverse ethnic backgrounds, they were all Canadian residents. Results could potentially be slightly biased by this factor, *additional studies* are necessary to identify the impacts of cultural backgrounds or country of residence in this type of analysis. In spite of that, this is the first attempt in the literature to account for a controlled environment to really understand gait and aspects driving performance figures in a gait-based biometric system. This additional analysis is vital to verify the impact of external and environmental factors to learn and generalize the established separability patterns between same-subject and different-subject feature vectors.

## Methods

### Multi-sensor gait dataset

The multi-sensor human gait dataset^24^ was adopted to analyze the reliability and generalization of gait recognition. This dataset comprises data from 25 subjects walking in a straight-level walkway with 42 reflexive markers over their body and a smartphone and a microcontroller device with an accelerometer attached to their legs. Subjects were balanced in gender identity (12 male, 12 female, 1 non-binary), and were aged between 18 and 47 years-old. Additional biometric data like height and weight was not collected. The study was advertised via e-mail to students and staff from McGill’s Schulich School of Music, which comprises persons from diverse ethnic backgrounds. Data were recorded on two different days, being captured ten trials in each day. All subjects walked barefoot across the same path in a controlled capture laboratory, using tight-fitting outfits to prevent any external or environmental factors from affecting the further analysis. Santos et al.^24^ processed the trials by gap-filling the trajectories and generating walking synchronized sessions of all sensors through Qualisys Track Manager (QTM) software. These processed walking sessions were used in this work. This dataset is publicly available on Figshare (https://doi.org/10.6084/m9.figshare.14727231.v6). QTM is a proprietary software, and requires a license to use. In this study, it was used a QTM license acquired by McGill University.

### Ethical statement

McGill University’s Research Ethics Board Office approved the acquisition of the multi-sensor human gait dataset and this research study (REB File # 198-1019). The data acquisition and this study was performed in agreement with the Helsinki declaration and its former amendments. Also, the data capture was conducted in accordance to the McGill University Policy on the Ethical Conduct of Research Involving Human Participants, and the Canada’s Tri-Council Policy Statement: Ethical Conduct for Research Involving Humans. All participants provided signed informed consent prior to entering the study.

### Data processing and analysis

The proposed investigations were accomplished using trajectories of the leg markers and MCU’s accelerometer readings intending to discuss topics related to gait analysis. The trajectories and accelerations of walking sessions extracted from trials were pre-processed by being split into frames using a sliding window of 256 samples and overlap of 75%.

Trajectories and accelerations have three axes, considering the cartesian coordinate system: *x* and *y* being two horizontal directions that compose a horizontal plane, and *z* being the vertical direction. These axes were aligned during the calibration of systems, as described by Santos et al.^24^. In the case of trajectories, the *x*-axis captures only the subject displacement. In the case of both trajectories and accelerations, the *z*-axis captures the patterns of body force exerted on the ground, whereas the *y*-axis denotates the lateral movement, which works as compensation of *z*-axis impulses to keep the resulting force aligned to the hip^33^. Thus, *z*-axis is the one that contains more critical information about gait cycles, events, and biomechanical movements. Therefore, only the *z*-axis of trajectories and accelerations were considered for the investigations.

Discrete Fourier Transform (DFT) coefficients of each frame *z*-axis were obtained, and the magnitude of those coefficients related to the frequencies higher and equal to 0 Hz (half of the coefficients because of its symmetry) were used as feature vectors. DFT coefficients were calculated using the Numpy FFT module (https://numpy.org/doc/stable/reference/routines.fft.html). This is an implementation of Fast Fourier Transform algorithm^34^ proposed to determine the signal DFT in a more efficient way than computing by it directly by its definition.

Fundamental frequencies were calculated using Christian’s Python Library (https://homepage.univie.ac.at/christian.herbst/python/namespacedsp_util.html), which applies an autocorrelation method based on Praat’s autocorrelation function. Given that the signals were split into frames during pre-processing stage, the function calculateF0once() was preferred as it is faster than the calculateF0() one.

Filtering processes were mainly handled by the Scipy Signal Processing Python module (https://docs.scipy.org/doc/scipy/reference/signal.html). The function scipy.signal.butter was employed to design Butterworth low-pass, high-pass and band-pass filters. The band-stop filter was designed using the pole-zero placement method^35^. All the designed filters were applied to the trajectories and accelerations using the Scipy Signal Processing function scipy.signal.lfilter.

The dimension of obtained feature vectors were reduced using UMAP to analyze the manifolds by basic visualization methods, like scatter plots. The official implementation of UMAP in Python was used^25^ (https://github.com/lmcinnes/umap). The reduced dimensionality space was set as two aiming to generate more understandable visualizations.

## Supplementary Information

Supplementary Information.

## Data Availability

The Python codes to perform the analysis are freely available on the first author’s github repository (https://github.com/geisekss/gait_reliability_investigation).
