# Supplementary material for: Reliability and generalization of gait biometrics using 3D inertial sensor data and 3D optical system trajectories
Source: Sci Rep. 2022 May 19;12:8414. doi: 10.1038/s41598-022-12452-6 (PMC9120026; doi:10.1038/s41598-022-12452-6)
Supplement: Supplementary file 1 — Supplementary Information. [file 41598_2022_12452_MOESM1_ESM.pdf]

Supplementary Information

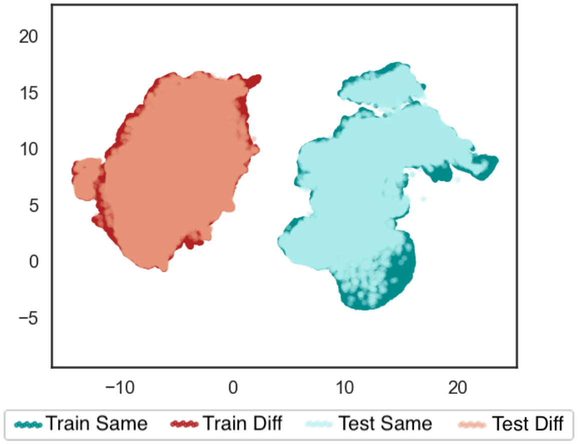

(a) Projections of feature vector pairs generated from accelerations. The training pairs were generated using the first random subset of 13 individuals.

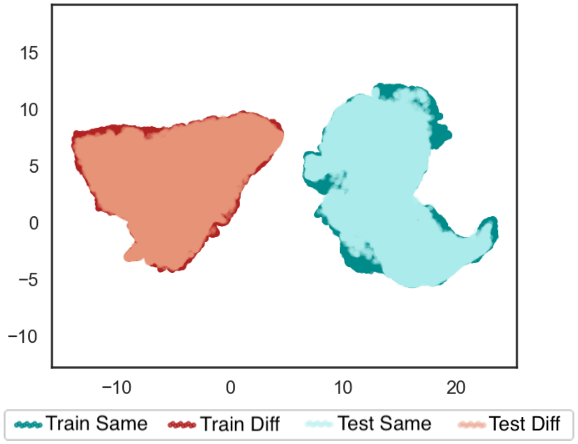

(b) Projections of feature vector pairs generated from accelerations. The training pairs were generated using the second random subset of 13 individuals.

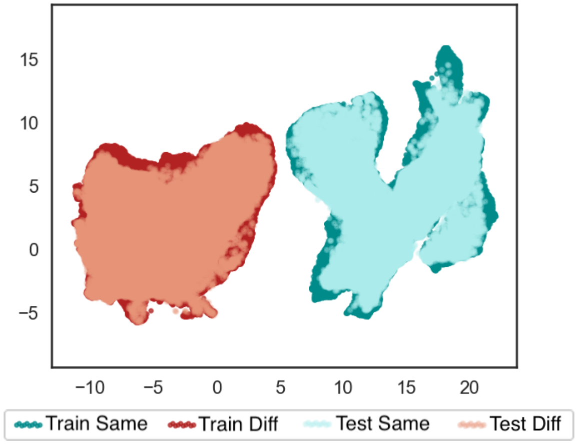

(c) Projections of feature vector pairs generated from accelerations. The training pairs were generated using the third random subset of 13 individuals.

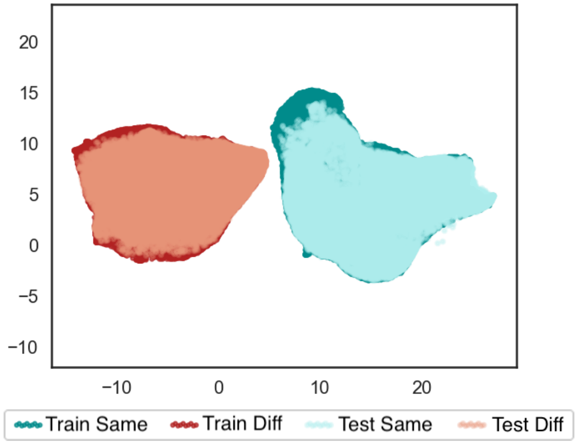

(d) Projections of feature vector pairs generated from `fle` trajectories. The training pairs were generated using the first random subset of 13 individuals.

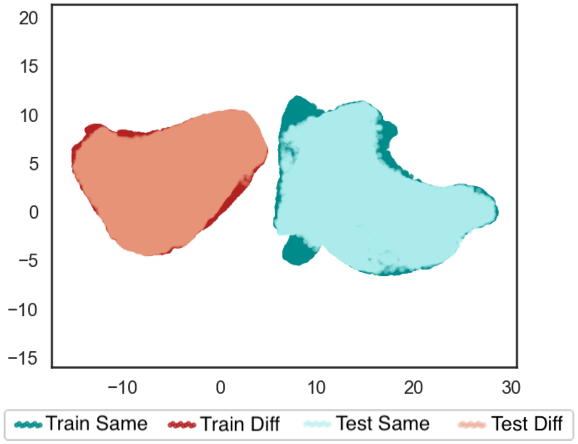

(e) Projections of feature vector pairs generated from `fle` trajectories. The training pairs were generated using the second random subset of 13 individuals.

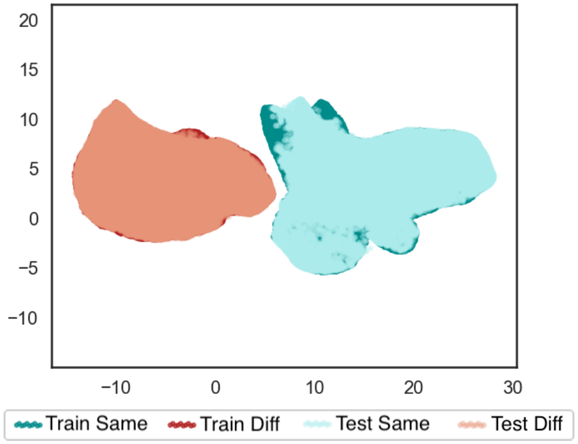

(f) Projections of feature vector pairs generated from `fle` trajectories. The training pairs were generated using the third random subset of 13 individuals.

Supplementary Figure S1. Bi-dimensional UMAP projections using the feature vector pairs generated using the accelerations and `fle` trajectories, after removing the DC component, from different random training sets of 13 individuals. For each experiment, the pairs were constructed using the random training set and the remaining individuals for test. Thus, four folds of pairs were generated: training set of same-subject pairs, training set of different-subjects pairs, test set of same-subject pairs, test-set of different-subjects pairs. The clusters generated from different training and testing sets are similar. This demonstrates low impact in changing the training subjects, specially for the `fle` trajectories, and good generalization capability of the learned relationships between pairs of same and different subjects.

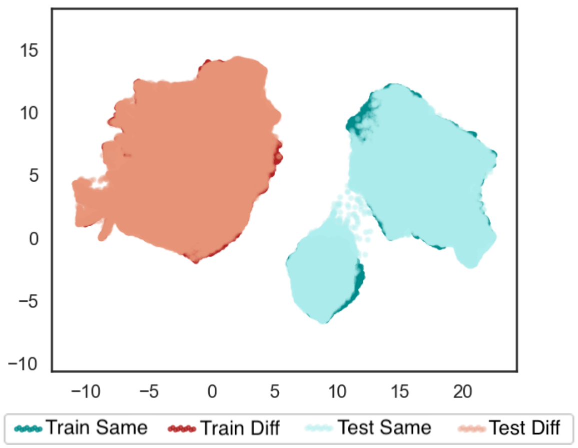

(a) Projections of feature vector pairs generated from accelerations without the DC component. The training pairs were generated using the first random subset of six individuals.

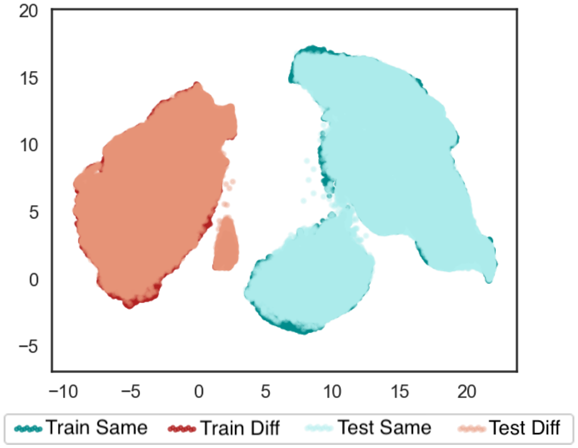

(b) Projections of feature vector pairs generated from accelerations without the DC component. The training pairs were generated using the second random subset of six individuals.

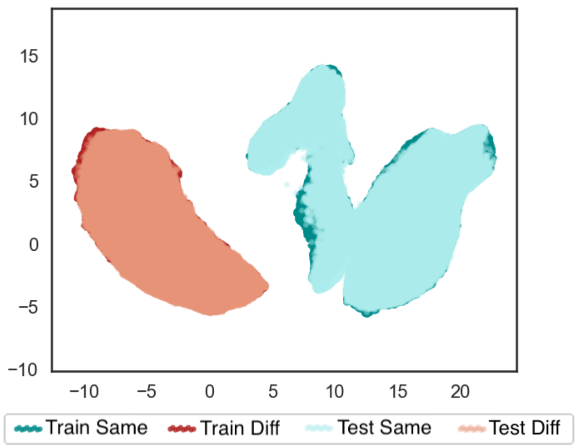

(c) Projections of feature vector pairs generated from accelerations without the DC component. The training pairs were generated using the third random subset of six individuals.

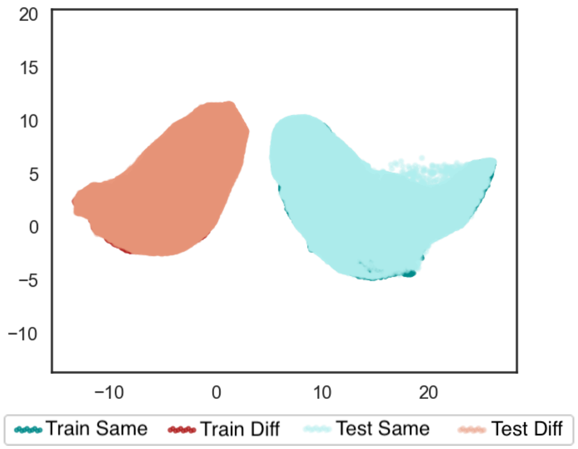

(d) Projections of feature vector pairs generated from fle trajectories without the DC component. The training pairs were generated using the first random subset of six individuals.

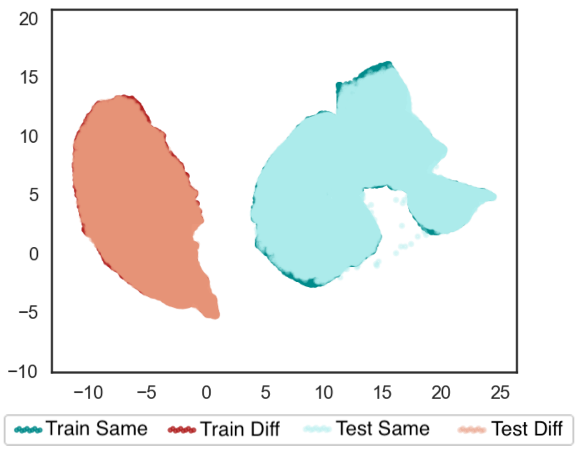

(e) Projections of feature vector pairs generated from fle trajectories without the DC component. The training pairs were generated using the second random subset of six individuals.

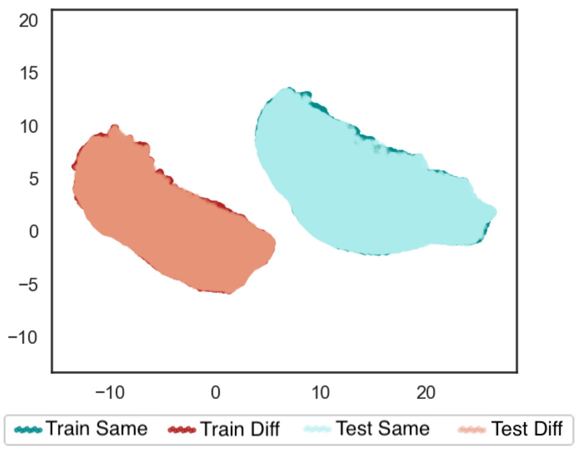

(f) Projections of feature vector pairs generated from fle trajectories without the DC component. The training pairs were generated using the third random subset of six individuals.

Supplementary Figure S2. Bi-dimensional UMAP projections using the feature vector pairs generated using different random training sets of six individuals. For each experiment, the pairs were constructed using the random training set and the remaining individuals for test. Thus, four folds of pairs were generated: training set of same-subject pairs, training set of different-subjects pairs, test set of same-subject pairs, test-set of different-subjects pairs. The clusters are less uniform according to each different training and testing set, mostly for the accelerations. In this case using only a training set with only six subjects, the chosen set of individuals has more impact than when using 13 subjects.

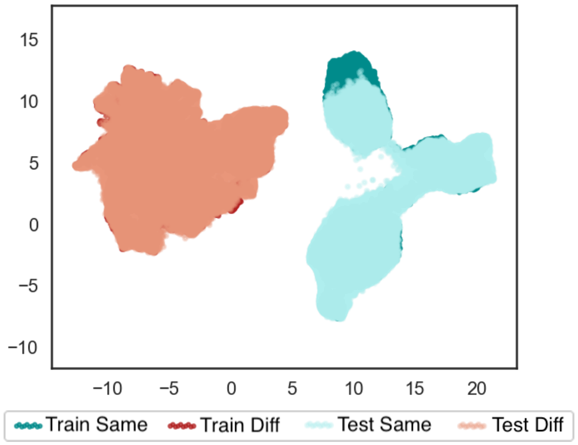

(a) Projections of feature vector pairs generated from accelerations without the DC component. The training pairs were generated using the first random subset of three individuals.

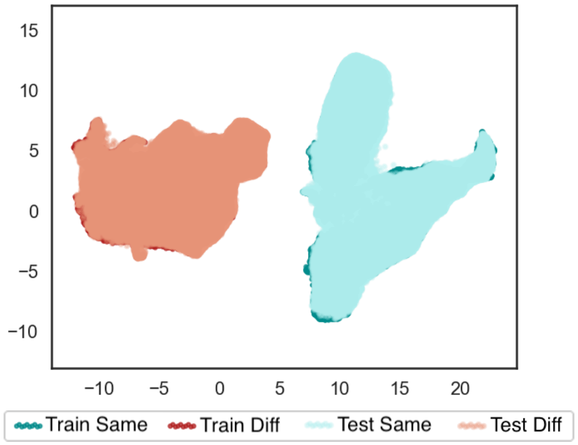

(b) Projections of feature vector pairs generated from accelerations without the DC component. The training pairs were generated using the second random subset of three individuals.

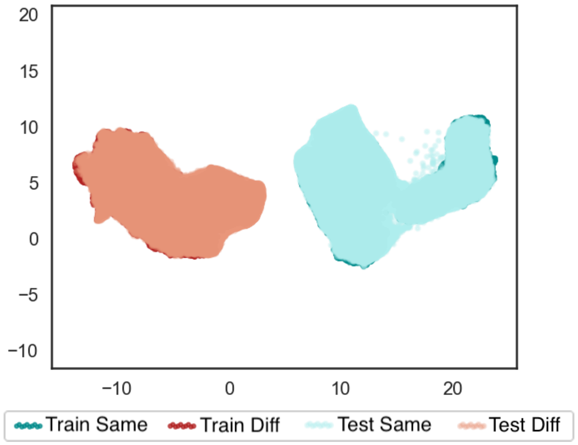

(c) Projections of feature vector pairs generated from accelerations without the DC component. The training pairs were generated using the third random subset of three individuals.

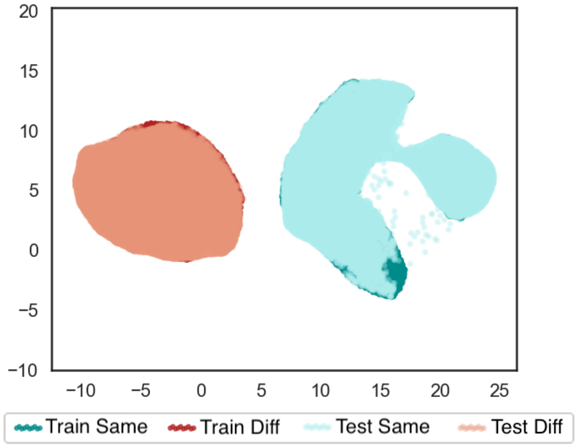

(d) Projections of feature vector pairs generated from fle trajectories without the DC component. The training pairs were generated using the first random subset of three individuals.

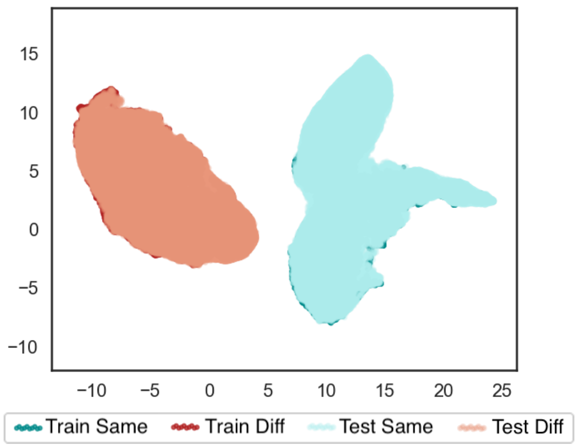

(e) Projections of feature vector pairs generated from fle trajectories without the DC component. The training pairs were generated using the second random subset of three individuals.

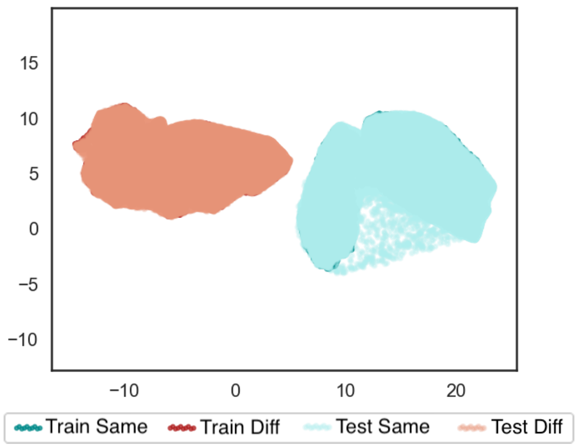

(f) Projections of feature vector pairs generated from fle trajectories without the DC component. The training pairs were generated using the third random subset of three individuals.

Supplementary Figure S3. Bi-dimensional UMAP projections using the feature vector pairs generated using different random training sets of three individuals. For each experiment, the pairs were constructed using the random training set and the remaining individuals for test. Thus, four folds of pairs were generated: training set of same-subject pairs, training set of different-subjects pairs, test set of same-subject pairs, test-set of different-subjects pairs. The clusters changes among the different sets of individuals for training and testing. These clusters also are more spread, breaking in smaller clusters. This demonstrates that reducing the number of individuals for training impacts the generalization capacity of the learned relationships between pairs of same and different subjects.
